# Highly efficient inoculum propagation in perfusion culture using WAVE Bioreactor™ systems

**DOI:** 10.1186/1753-6561-7-S6-P7

**Published:** 2013-12-04

**Authors:** Christian Kaisermayer, Jianjun Yang

**Affiliations:** 1 GE Healthcare Life Sciences, Björkgatan 30, 751 84 Uppsala, Sweden; 2 GE China Research and Development Center Co. Ltd. Shanghai, China

## Introduction

A perfusion-based process was developed to increase the split ratio during the scale-up of CHO-S™ cell cultures. Fedbatch cultures were inoculated with cells propagated in either batch or perfusion cultures. All cultures were grown in disposable Cellbag™ bioreactors using the WAVE Bioreactor system. Cell concentrations of 4.8 × 10^7 ^cells/mL were achieved in the perfusion culture, whereas the final cell concentration in the batch culture was 5.1 × 10^6 ^cells/mL. The higher cell concentration of the perfusion culture allowed for a more than six-fold increase of the split ratio to about 1:30. The method described here, can reduce the number of required expansion steps and eliminate the need for one or two bioreactors in the seed train. Single-use bioreactors at benchtop scale can be used for direct inoculation of production bioreactors. Alternatively, high biomass concentrations accumulated in perfusion culture can be used to seed production vessels at increased cell concentrations. Thus, the process time in these bioreactors, which often is the bottleneck in plant throughput, can be shortened.

## Materials and methods

• CHO-S cells (Life Technlologies)

• Cultivation medium and feed concentrate: T13 and T13-F (Shanghai Hankang Biotech Co.)

• WAVE Biorereactor 20/50 system (GE Healthcare)

• Cellbag bioreactors (GE Healthcare)

Batch and fed-batch cultivations were run in Cellbag 10 L bioreactors, perfusion cultures in Cellbag 2 L bioreactors. Cultivation conditions: T 37°C, pH 7.10, DO > 40%, agitation for all cultures 25 rpm/6°.

Analytics: Cell concentration and viability, glucose and lactate concentration. Perfusion and feed rates were adjusted to maintain the residual glucose concentration above 0.5 g/L.

## Results and discussion

**Figure 1:**
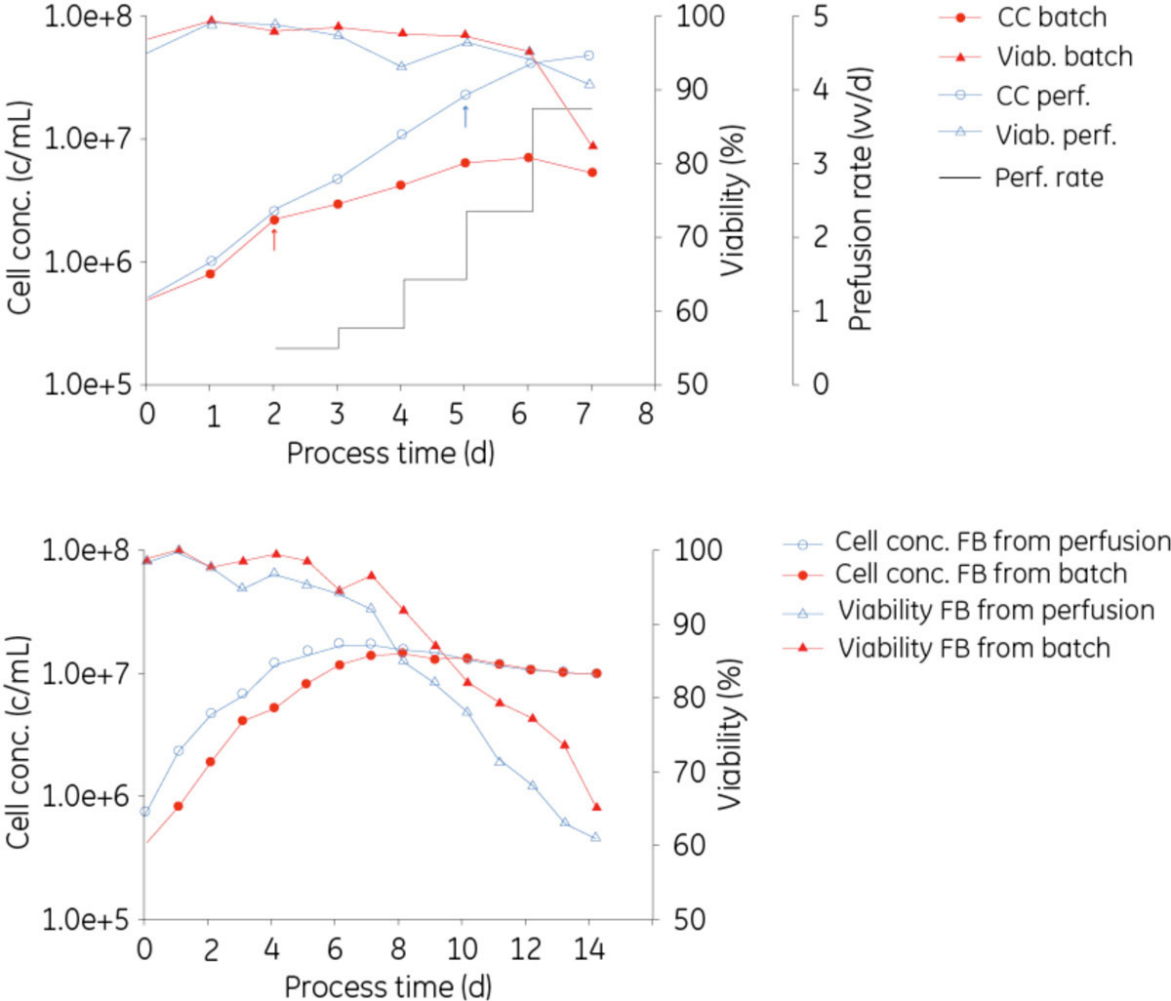
**CHO-S cells grown in batch and perfusion**. Arrow indicates seed removal for subsequent fedbatch cultures (upper panel). Comparison of CHO-S fed-batch cultures inoculated from either batch or perfusion (lower panel).

CHO cells are the production system of choice for complex recombinant proteins. The prevalent mode of production is fedbatch cultivation because of the generated titers achieved with limited process complexity [1]. Perfusion processes have been reported as an alternative strategy that substantially increases volumetric productivity but because of the higher process complexity, they are less frequently used in manufacturing [2,3]. An alternative strategy is to use perfusion technology in the seed train to improve process flexibility and maximize equipment utilization [3]. In this comparative study, CHO-S cells were grown in either batch or perfusion (Figure 1) culture to generate inocula for subsequent fed-batch cultivations. During the initial phase, cell growth in both cultures was similar (Figure 1). However, despite high cell viability, the growth rate in the batch culture decreased from 0.8 d^-1 ^during the first two days to about 0.3 d^-1 ^between day 2 and 6 (data not shown). In contrast, the nutrient supply in the perfusion culture supported an average growth rate of 0.8 d^-1 ^and an exponential growth until day 5 (Figure 1). Inoculum was removed from each seed culture while the cells were still growing at their maximum rate and while viability was above 95%. The higher cell concentration achieved in the perfusion culture was used to seed a subsequent fedbatch culture at an increased split ratio of 1:30, compared with 1:5 used for the inoculum from the batch culture. Cell growth in the two subsequent fed-batch cultures is shown in Figure 1. The cultures inoculated from either batch or perfusion culture showed comparable growth and no lag phase was observed after inoculation. A comparison of the individual culture parameters is presented in Table 1. The higher split ratio in the perfusion culture saves at least one step in the inoculum propagation as compared with cultivation in batch mode, for which two subsequent cultures with a split ratio of 1:5 would be required to obtain a similar ratio. Even higher split ratios could be achieved in perfusion cultures. On day 6, the cell concentration was 4.06 × 10^7 ^cells/mL with a viability of 96%. (Figure 1). Although the cells were already at the end of the exponential growth phase, a split ratio of 1:100 could be achieved at this timepoint. The fed-batch culture inoculated from the perfusion culture was started at a substantially higher cell concentration than the one inoculated from the batch culture and, thus, reached its maximum cell concentration about two days earlier (Figure 1). Additionally the viable cell integral was increased by about 20% (data not shown). Assuming constant product formation during cell growth, this would allow to reach the same amount of product two days earlier and, thus, shorten process time in the main bioreactor. The use of perfusion cultures for seeding the production bioreactor at high cell concentrations has also been reported for an industry process at 13,500 L working volume where it resulted in a 20% decrease in the occupation of the production vessel [3].

**Table 1:** Comparison of fed-batch cultures

	FB seeded from batch	FB seeded from perfusion
Cell conc. at cell removal [c/mL]	2.2 × 10^6^	2.3 × 10^7^
Split ratio	1:5	1:30

Inoculum conc. [c/mL]	4.1 × 10^5^	7.4 × 10^5^

Process time [d]	14	14

Peak cell conc. [c/mL]	1.4 × 10^7^	1.7 × 10^7^

Av. μ during growth phase [d^-1^]	0.44	0.52

Inoculum propagated either in batch or perfusion culture

## Conclusions

• Perfusion culture maintained cells in exponential growth phase for an extended period of time compared with batch culture.

• The high cell concentrations obtained in perfusion culture can substantially increase the split ratio, thus, minimizing the number of vessels needed in the seed train.

• Alternatively, the production bioreactor can be inoculated at high cell concentration, which can help shortening process time in the production vessel and improving facility utilization.

• One WAVE Bioreactor 20/50 system, run in perfusion mode at the maximum operating volume of 25 L, could provide inoculum for a 2000 L bioreactor.
